# Pairwise growth competitions identify relative fitness relationships among artemisinin resistant *Plasmodium falciparum* field isolates

**DOI:** 10.1186/s12936-019-2934-4

**Published:** 2019-08-28

**Authors:** Abigail R. Tirrell, Katelyn M. Vendrely, Lisa A. Checkley, Sage Z. Davis, Marina McDew-White, Ian H. Cheeseman, Ashley M. Vaughan, François H. Nosten, Timothy J. C. Anderson, Michael T. Ferdig

**Affiliations:** 1 Eck Institute for Global Health, Dept. of Biological Sciences, University of Notre Dame, Notre Dame, IN USA; 2 Texas Biomedical Research Institute, San Antonio, TX USA; 3 Seattle Children’s Research Institute, Seattle, WA USA; 4 Shoklo Malaria Research Unit, Mahidol-Oxford Tropical Medicine Research Unit, Mahidol University, Mae Sot, Thailand; 5 Centre for Tropical Medicine and Global Health, Nuffield Department of Medicine Research Building, University of Oxford Old Road Campus, Oxford, UK

**Keywords:** Competitive growth, Fitness, Artemisinin resistance, *kelch13*, Population genetics

## Abstract

**Background:**

Competitive outcomes between co-infecting malaria parasite lines can reveal fitness disparities in blood stage growth. Blood stage fitness costs often accompany the evolution of drug resistance, with the expectation that relatively fitter parasites will be more likely to spread in populations. With the recent emergence of artemisinin resistance, it is important to understand the relative competitive fitness of the metabolically active asexual blood stage parasites. Genetically distinct drug resistant parasite clones with independently evolved sets of mutations are likely to vary in asexual proliferation rate, contributing to their chance of transmission to the mosquito vector.

**Methods:**

An optimized in vitro 96-well plate-based protocol was used to quantitatively measure-head-to-head competitive fitness during blood stage development between seven genetically distinct field isolates from a hotspot of emerging artemisinin resistance and the laboratory strain, NF54. These field isolates were isolated from patients in Southeast Asia carrying different alleles of *kelch13* and included both artemisinin-sensitive and artemisinin-resistant isolates. Fluorescent labeled microsatellite markers were used to track the relative densities of each parasite throughout the co-growth period of 14–60 days. All-on-all competitions were conducted for the panel of eight parasite lines (28 pairwise competitions) to determine their quantitative competitive fitness relationships.

**Results:**

Twenty-eight pairwise competitive growth outcomes allowed for an unambiguous ranking among a set of seven genetically distinct parasite lines isolated from patients in Southeast Asia displaying a range of both *kelch13* alleles and clinical clearance times and a laboratory strain, NF54. This comprehensive series of assays established the growth relationships among the eight parasite lines. Interestingly, a clinically artemisinin resistant parasite line that carries the wild-type form of *kelch13* outcompeted all other parasites in this study. Furthermore, a *kelch13* mutant line (E252Q) was competitively more fit without drug than lines with other resistance-associated *kelch13* alleles, including the C580Y allele that has expanded to high frequencies under drug pressure in Southeast Asian resistant populations.

**Conclusions:**

This optimized competitive growth assay can be employed for assessment of relative growth as an index of fitness during the asexual blood stage growth between natural lines carrying different genetic variants associated with artemisinin resistance. Improved understanding of the fitness costs of different parasites proliferating in human blood and the role different resistance mutations play in the context of specific genetic backgrounds will contribute to an understanding of the potential for specific mutations to spread in populations, with the potential to inform targeted strategies for malaria therapy.

## Background

A drug resistance-associated mutation that confers a fitness advantage in the presence of drug often imposes a fitness cost in the absence of drug pressure [1]. Fitness costs can manifest as reduced in-host growth rate that can lead to declining frequencies of drug-resistant pathogens in populations when drug pressure is removed [2].

Competitive growth is one indicator of the relative fitness of co-infecting parasite lines. For pathogens including HIV, fungi, and bacteria, the gold standard for measuring fitness in vitro and in vivo relies on labeling methods to assay the relative growth success of the competing lines [3]. While both cooperative and competitive interactions can occur between co-infecting conspecific pathogens [4–6] head-to-head growth outcomes usually reflect the innate proliferation rates of each of the competing genotypes.

Competitive growth assays have been used to determine the fitness relationships among drug-resistant *Plasmodium falciparum* lines. For example, a quantitative PCR method was used to track the interactions and competitive outcomes of mixed infections containing a chloroquine-sensitive (CQ-S) and a CQ-resistant (CQ-R) line in the absence of CQ; the CQ-S parasite line consistently outcompeted the CQ-R line [7–12]. Clinical artemisinin resistance (Art-R), manifests as a delayed clearance of parasites from a patient’s blood following drug treatment and is defined as a parasite clearance half-life ≥ than 5 h after drug treatment [13]. Parasites isolated from these patients have been studied using competitive growth assays [14] and a few focused laboratory experiments have demonstrated a cost of Art-R mutations in *P. falciparum* lines grown in the absence of drug [15, 16].

The broad diversity of the genetic variants associated with Art-R and their relative effects on fitness have not been thoroughly investigated. The delayed clearance phenotype (clinical Art-R) is associated with single-nucleotide polymorphisms (SNPs) encoded by *kelch13* [17]. As of 2016, 124 independent non-synonymous substitutions in *kelch13* had been identified: 46 mutations unique to Southeast Asia, 62 found in sub-Saharan Africa, and 16 found in both regions. Not all of these mutations have been associated with clinical resistance; only those mutations found in Southeast Asia or both regions have been linked, in vivo or in vitro, to resistance-related phenotypes. These mutations typically encode changes in the propeller domain of the protein coded by *kelch13* [18, 19].

In spite of the many mutations in *kelch13* and independent origins of Art-R in Southeast Asia, only one of these mutations (C580Y) is expanding in frequency and replacing other *kelch13* mutations along the Thailand–Myanmar border and in Cambodia, Vietnam, and southern Laos [13, 20–22]. Notably, some Art-R parasites on the Thailand–Myanmar border and in Cambodia do not carry coding mutations in *kelch13*, indicating that factors other than, and/or in addition to, altered *kelch13* contribute to this resistance phenotype [23, 24]. Technology advances are allowing researchers to monitor and observe the emergence and spread of Art-R in real time, an approach that was not available for tracking the worldwide sweep of CQ-R from a few independent origins decades before. Commensurate with this capacity to observe emerging drug resistance is the opportunity to anticipate and act to limit the devastation that would ensue if artemisinin-based drug combination therapies fail worldwide. Understanding the genetic basis of relative fitness and propensities of different forms of Art-R parasites to spread will be one component of a multi-pronged strategy to retain this valuable drug that has in part, underpinned the remarkable decline in malaria deaths over the past 15 years.

To date, experimental competitive growth studies of *Plasmodium* parasites in vitro have been limited to a few pairwise competitions to discern growth relationships among a few (two or three) parasite lines [7, 8, 15, 16] due to the volume requirement required to determine the ratio of the competing parasite lines. Typically, the ratio of each line in an in vitro competition assay is determined by pyrosequencing [16, 25] amplicon sequencing [26], or quantitative PCR [7, 8, 16, 27]. These methods are accurate and can easily distinguish competing parasite lines, however they can be cumbersome, time consuming, and costly. These methods require relatively large volumes of either a DNA template, usually obtained via a blood DNA extraction, or enough parasitized red blood cells for analysis, which requires a large culture volume. Decreasing the volume of culture needed in order to distinguish between competing parasite lines would allow for a much higher throughput and require less time and reagents.

This optimized method uses straight-from-blood PCR for microsatellite genotyping; increasing the throughput and efficiency of competitive growth assays compared to previous methods. Here, a 96-well plate-based in vitro competitive growth assay is used to compare eight parasite lines (28 head-to-head competitions) to quantify the relative fitness relationships of a series of field-isolated Art-R and Art-sensitive lines. This tool expands the capacity to precisely catalogue fitness relationships that can facilitate a broader genetic-based approach to understanding, predicting, and preventing the spread of drug resistance.

## Methods

### Parasite culture

Cryopreserved stocks of cloned parasite lines were thawed and grown in complete media (CM) [0.5% Albumax II (Gibco), 10 μg/ml gentamycin (Gibco), 7.5% sodium bicarbonate (Corning), and incomplete media (ICM) (Gibco)] at 5% haematocrit in O^+^ red blood cells (RBC). Cultures were maintained at constant pH, 7.0–7.5, temperature, 37°, and atmosphere, 5% CO_2_/5% O_2_/90% N_2_. Cultures were kept below 3% parasitaemia with media changes every generation of the intraerythrocytic development cycle (IDC) (48 h).

### Life cycle synchronization

Parasites were triple synchronized using 5% d-sorbitol. Initial synchronization occurred when most of the parasites in culture were in the early stages of the life cycle. 48 h later the second synchronization was performed, with the third synchronization occurring 8 h later. Parasites were considered synchronized when at least 80% of the parasites in culture were in the early trophozoite (ring) stage of the life cycle.

### Ring-stage survival assay as an in vitro metric to confirm resistant isolates

The ring-stage survival assays from 0 to 3 h were carried out as previously described with minor modifications [28]. Briefly, parasites were grown to 40 ml cultures at 5% hematocrit and schizonts were synchronized using a MACS magnet column (Miltenyi Biotec) into 10 ml cultures. Once schizonts had burst and merozoites had reinvaded, 2 ml of culture was transferred to 6 wells of 12-well cell culture cluster plates (Corning) at 2% hematocrit and 2% parasitaemia. Half of the wells were treated with 700 nM dihydroartemisinin (DHA) (Sigma) and the other half of the wells were treated with dimethyl sulfoxide (DMSO) (ThermoFisher). After 6 h all cultures were washed with ICM three times and transferred to a new plate to ensure complete removal of the drug. After an additional 66 h, slides were made on all cultures and 5000 RBCs were counted per culture. Proliferation was measured by the percent parasitaemia in the DHA treated culture over the percent parasitaemia in DMSO treated cultures. Two biological replicates each with three technical replicates were carried out for NHP4026, NHP4076, NHP4333, NHP1337, and NF54. Parasites are considered resistant if the percent proliferation is greater than 5% [13].

### Optimization of competitive growth assays

48 h following the final synchronization, a series of competitions was designed among five genetically distinct parasite lines (NHP4026, NF54, P1, P2, and P3) to compare the standard 5 ml flask-based method and the optimized 96-well plate-based method. Each competition was established in both formats (culture flasks at 5 ml volumes and in 96-well plates, each well with a total volume of 200 μl), each with three technical replicates. Each competition consisted of two genetically distinct parasite lines each at 0.5% parasitaemia for a total of 1% parasitaemia at the start of the assay. Every IDC, cultures were diluted to 1% parasitaemia in the 5 ml flasks, or to 50 μl volume in the 96-well plate repetitions, and fresh RBCs and media were added. Samples from each culture were collected and stored at − 80 °C at the time of each dilution. Every other IDC, samples from the 96-well plate competitions were fixed and stained with Giemsa (Sigma) to estimate parasitaemia via microscopy.

### Microsatellite marker development

Twenty-two of the microsatellite markers (MS) first described in the high-resolution linkage map for *P. falciparum* [29] were selected to be evaluated as described previously [30] to differentiate between the various isolates. Briefly, fluorescently labeled primers specific to the 22 MS [30] distributed across the 14 chromosomes of *P. falciparum* were evaluated on the set of eight parasite lines to arrive at an optimized set of four MS that could differentiate between each of the 28 combinations of the parasite lines. Microsatellites were selected to differentiate between two lines if the difference between the two fragment sizes was five base pairs or greater. A majority of the parasite lines were able to be differentiated using two of the MS (TA119 and TA81), however the two sets of parasite lines that could not be differentiated using these two MS were differentiated with two different MS (TA77 and TA62) (Additional file 1).

These 22 MS could be optimized to differentiate diverse parasite lines, however the number of MS needed depends on the parasite lines as well as the resolution required (the minimum detectable difference between the fragments differentiating parasite lines).

### Amplification and Capillary Electrophoretic Genetic Analysis System (CEQ) fragment analysis of microsatellite markers

Samples were collected at 48 h intervals throughout the duration of the assay. To determine relative densities of each parasite, four microsatellite markers designed to generate distinct fragment sizes for each parasite line were used for PCR amplification using the Phusion Blood Direct PCR Kit (ThermoFisher, cat # F547L); primers were labelled with Well-Red fluorescent dyes (Sigma, custom order). Annealing temperatures were determined using the ThermoFisher Tm Calculator. Reactions were set up at 20 μl. Thermocycler conditions were as follows: denaturation for 5 min at 98 °C, followed by 30 cycles of 98 °C for 1 s, optimal annealing temperature for 5 s, and 65 °C for 15 s. Final extension was at 65 °C for 1 min (Additional file 1). Amplified samples were analysed through fragment analysis using the CEQ 8000 Genetic Analysis System (Beckman Coulter). Quantitative fragment analysis was used to determine the relative densities of each parasite in the competition by scoring the fluorescent peak height of the corresponding PCR product size of each parasite as a proportion of the overall signal. Resulting proportions from each sampling day were plotted using GraphPad Prism 6.0. CEQ fragment analysis of marker densities was used to compare the outcomes from the standard 5 ml culture method to outcomes from the 96-well plate method.

To validate the method of parasite-line specific PCR product density detection, DNA extracted from two different lines was quantified using the Qubit 2.0 Fluorometer system (ThermoFisher) and ratios were established to generate a standard curve. Samples were then amplified using specific microsatellite markers primer sets (Additional file 2).

### Parasite lines

Seven genetically distinct parasite lines, both sensitive and resistant to artemisinin as defined by their parasite clearance half-life were isolated from patients with hyper-parasitaemia (> 4%) in Southeast Asia carrying different *kelch13* alleles. These lines were derived from cloning by limiting dilution from patient blood, including an artemisinin-sensitive *kelch13* wild-type (wt) line (NHP4302), an artemisinin-sensitive line with a mutation in *kelch13* (NHP3032, *kelch13* K438N), three lines with delayed clearance rates (NHP4333, NHP1337, and NHP4076) with mutations in *kelch13* (G538V, C580Y, and E252Q, respectively), and two lines with delayed clearance rates but without coding mutations in *kelch13* (NHP4026 and NHP4373). All seven lines were isolated from patients on the Thailand–Myanmar border between 2008 and 2011. An eighth line, drug-sensitive NF54, was used as a growth control for comparison to the seven Southeast Asian lines (Table 1).

**Table 1:** Parasite lines selected for inclusion in the competition assay

Parasite	*kelch13* genotype	Artemisinin clearance rate	Parasite clearance half-life (*t*_*1/2*_*P*) [31]	Isolate collection date [32, 33]	Location [32–34]
NF54	Wild-type	Fast	–	–	–
NHP4302	Wild-type	Fast	1.98	17-Sept-08	Wang Pha
NHP3032	K438N	Fast	2.17	14-Jul-08	Maela
NHP4026	Wild-type	Slow	8.37	07-Dec-07	Wang Pha
NHP4373	Wild-type	Slow	7.10	15-Dec-08	Wang Pha
NHP4333	G538V	Slow	7.77	29-Oct-08	Wang Pha
NHP1337	C580Y	Slow	7.84	19-Apr-11	Mawker Thai
NHP4076	E252Q	Slow	5.03	6-Feb-08	Wang Pha

Eight parasites were included in the competitive growth assays. Each line is designated as *kelch13* wild-type or mutant sequence, either as a fast clearer (artemisinin-sensitive) or slow clearer (artemisinin-resistant) in patients. In addition, parasite clearance half-life [31], and the date and location for each line is shown [32–34]

### Competitive growth assessments

Using the 96-well competitive growth method described above, each of the eight parasite lines were co-cultured in pairwise competitions with all others in three technical replicates and maintained for 14–60 days. Four biological replicates each with three technical replicates were carried out for the NF54 versus NHP4026 competition (Additional file 3), which determined that the outcome of the competition was consistent despite different starting ratios of each parasite (day 0) and the day in which one parasite line reached 95% of the population, ending the competition. Because the phenotype of interest is the identity of the winning parasite line (regardless of the duration of time), only one biological replicate with three technical replicates was completed for the remainder of the competitions. At 48-h intervals, parasitaemia was assessed and diluted as described, samples were collected, and microsatellite markers were amplified. Fragment analysis was performed to determine relative densities of each parasite in the mixed cultures at each time of collection.

### Duration of assays and determination of winners and losers

Competition assays were continued until one parasite line was determined to compose ≥ 95% of all parasites in the culture. Competitions were stopped at 60 d if neither outcompeted the other. Winners were determined at the conclusion of each competition assay. A complete win resulted if one line fully outcompeted the other (≥ 95% of the total parasitaemia by day 60). A partial win was defined as one line stabilizing at ≥ 70% of the total parasitaemia by day 60. Both complete and partial wins were considered wins. Each parasite line’s win/loss record was used to rank the eight parasite lines in a hierarchy from most competitively fit (only wins) to least fit (only losses).

### Timeline of optimized competitive growth assays

The straight-from-blood PCR kit used in combination with the MS to generate distinct fragment sizes for each parasite line allowed for a dramatically reduced culture volume and reduced the time needed to complete the entirety of the assay (Additional file 4). Cryopreserved stocks of all eight parasite lines were thawed (which took about 2 h) and only grown to 10 ml of culture, which took approximately two life cycles. Methods that require greater volumes of culture require several more life cycles to grow up a larger volume at an appropriate parasitaemia. When cultures were approximately 2% at 10 ml, all cultures were triple synchronized using 5% d-sorbitol (immediately, and then 48 h after the first synchronization, and finally at 56 h after the first synchronization). Cultures were left for one life cycle and then parasitaemia was counted and all cultures were set up in the 96-well plate with three technical replicates (28 all-on-all competitions of eight parasite lines with three technical replicates was 84 wells in a 96-well plate). The total time from thawing to assay initiation was approximately 10 days. Alternatively, the remaining culture (not used to set up the first biological replicate) can be grown for an additional cell cycle and set up in the 96-well plates with three technical replicates as long as the culture remains synchronized (at least 80% of the parasites in culture were in the early ring stage of the life cycle); this accommodates biological replicates in the study design.

After competitive growth assays were set up in 96-well plates, they were maintained in the plate for 20–60 days (until one parasite line reached 95% of the population). Ninety-six-well format is simpler, faster and requires less space than flask methods. To differentiate between the two competing parasite lines, samples were collected from diluting the parasitaemia each life cycle and stored at − 80 °C until they were analysed. To increase efficiency, samples were analysed in large groups every 10 days to monitor the progress of each competition (samples were taken every 2 days, so 5 samples were analysed per competition from 28 competitions for a total of 140 samples every 10 days). Straight-from-blood PCR eliminated any need for DNA extractions, which saved time and greatly decreased the amount of whole culture needed for each PCR reaction (only 1 μl is needed). Setting up 140 reactions for PCR took approximately 1 h and running on a thermocycler took 1 h. Diluting the PCR and preparing the 140 samples to run on the CEQ took approximately 1 h. Samples were loaded and run on the CEQ (took about 8 h to run 140 samples); the run time depends on the number of samples and the specific fragment analysis machine being used. The fragment analysis of 140 samples took about 2 h. Altogether, the analysis from PCR to determining the relative ratios of each parasite line throughout the entirety of the competition took approximately 1 day.

## Results

### The 96-well plate format recapitulates the standard competition assay results

**Fig. 1:**
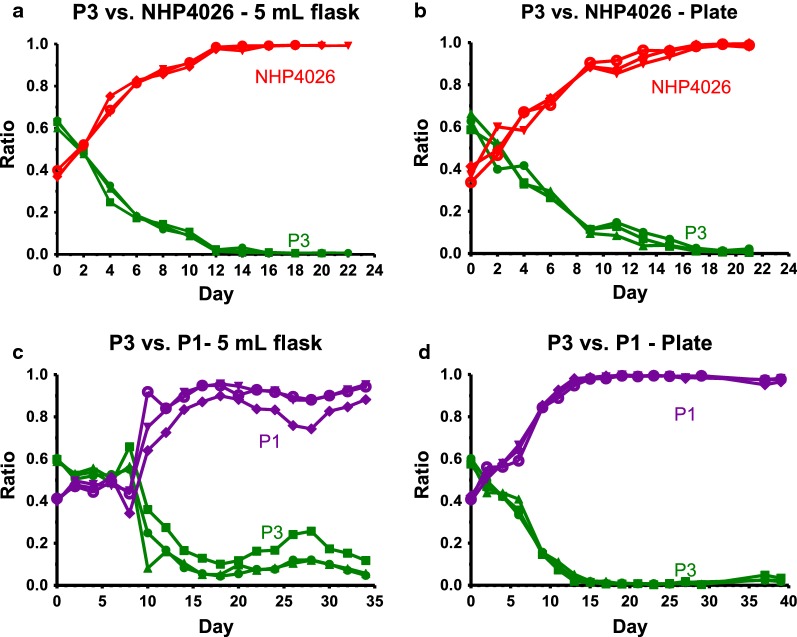
Consistent outcomes between 5 ml flask and 96-well plate methods in parasite competitive growth assays. Competition assays began with a parasite ratio of approximately 0.5:0.5. DNA microsatellite markers were used to track the competitive growth of parasites over time. Parasite competition P3 versus NHP4026 (n = 3) revealed the ability of NHP4026 to outcompete P3 by day 22 in both 5 ml flasks (**a**) and 96-well plates (**b**). The competition between P3 and P1 (n = 3) demonstrated the ability of P1 to outcompete P3 in 5 ml flasks by day 34 (**c**) and in 96-well plates by day 50 (**d**). Overall outcome results were consistent between 5 ml flask and 96-well plate, confirming the reliability of 96-well plate competitive growth assay methodology

Competitive growth assays for *P. falciparum* have been conducted previously in 5 ml culture flasks, requiring more time, space, and reagents than is suitable for large studies with many parasites [7, 26, 35]. To optimize a higher-throughput method to ascertain the relative fitness of many parasite lines, the previous protocol was adapted and optimized to a 96-well plate format and compared it to outcomes from standard 5 ml flask competition assays. Four pairwise competitions of genetically distinct lines derived from recent natural clinical patient isolates were conducted using both methods. For each of the four competitions, results were consistent across methods. Parasite NHP4026 outcompeted parasite 3 (P3) to compose over 95% of the culture (considered complete) by day 22 (n = 3; Fig. 1a, b). P1 completely outcompeted P3 in both the 5 ml flask and 96-well plate; however, in the 5 ml flask the competition went to completion by day 34, while in the 96-well plate it went to completion by day 50 (n = 3; Fig. 1c, d). P3 versus NF54 and P3 versus P2 also were consistent between experimental platforms (Additional file 5). Given the success of this validation experiment, all subsequent competitive growth assays were conducted using 96-well plates.

### Win/loss records rank lines by their competition outcomes

To comprehensively measure relative fitness, pairwise competitive growth assays were carried out for all eight lines; these clonal parasite lines were chosen to represent a range of *kelch13* mutations, clearance times and genetic backgrounds (Table 1). Each cloned line was competed with three technical replicates against each of the other seven cloned lines, in the absence of drug pressure, using the optimized 96-well plate methodology (Additional file 6).

A transitive relationship among the competition outcomes was observed; if line A outcompeted line B, and line B outcompeted line C, then A also outcompeted C, enabling unambiguous ranking of each parasite line. In this small set of parasites, all-on-all competitions were feasible. These data indicate that competitive outcomes of pairwise experiments from much larger parasite samples can be accurately predicted.

**Fig. 2:**
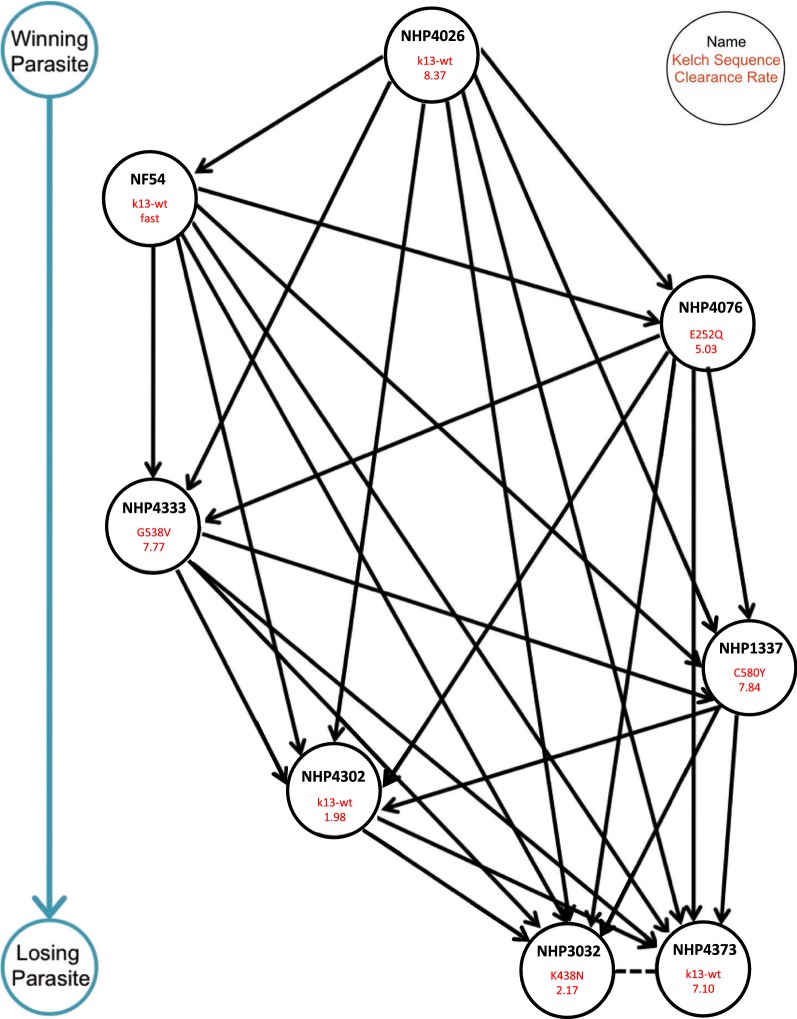
Win/loss records between parasite lines generate a fitness hierarchy. Win/loss records were determined for each parasite line and used to order lines from highest competitive fitness to lowest fitness. Arrows point from the winning (more fit) parasite to the losing (less fit) parasite. The win/loss record hierarchy ranked the *kelch13*-wt, slow clearance NHP4026 as having the highest fitness (7 wins, 0 losses: 7-0 record). Both NHP3032 and NHP4373 were unable to outcompete any other parasite, and the NHP3032 versus NHP4373 competition was unresolved, giving both isolates a record of 0 wins, 0 losses, 1 tie (0-0-1), suggesting potential fitness disadvantages in these isolates. NHP4302 had a record of 2-5. The hierarchy also shows that the *kelch13* mutant isolates, NHP1337 (3-4 record), NHP4333 (4-3 record), and NHP4076 (5-2 record) have middle-range fitness and have a fitness cost relative to the uncharacterized resistance in NHP4026 and to the sensitive lab line, NF54 (6-1 record)

The win/loss records established the relative fitness ‘rankings’ for all eight lines: NHP4026 (*kelch13*-wt, slow clearance) > NF54 (*kelch13*-wt, fast clearance) > NHP4076 (*kelch13* E252Q, slow clearance) > NHP4333 (*kelch13* G538V, slow clearance) > NHP1337 (*kelch13* C580Y, slow clearance) > NHP4302 (*kelch13*-wt, fast clearance) > NHP3032 (*kelch13* K438N, fast clearance) = NHP4373 (*kelch13*-wt, slow clearance) (Additional file 6). The most competitively advantaged (i.e. fit) lines (NHP4026 and NF54) always exhibited complete wins, the middle fitness lines (NHP4076 and NHP4333) demonstrated a mix of complete and partial wins and (NHP1337 and NHP4302) had only partial wins. The least fit lines (NHP3032 and NHP4373) did not have any wins (outcompeted no other line). Moreover, the NHP3032 versus NHP4373 competition was unresolved (i.e. a ‘tie’). Amongst the Southeast Asian lines, NHP4026, an Art-R line with *kelch13*-wt, exhibited the highest relative fitness, followed by NHP4333, NHP4333, and NHP4076, three further Art-R lines with *kelch13* mutations. A tie was observed between NHP4373, an Art-R line with *kelch13*-wt and NHP3032, an Art-sensitive line with the *kelch13* K438N mutation. This latter observation is consistent with these parasites having the lowest relative fitness among all eight parasite lines (Fig. 2).

### The uncharacterized Art-R line NHP4026 may confer a broad fitness advantage

**Fig. 3:**
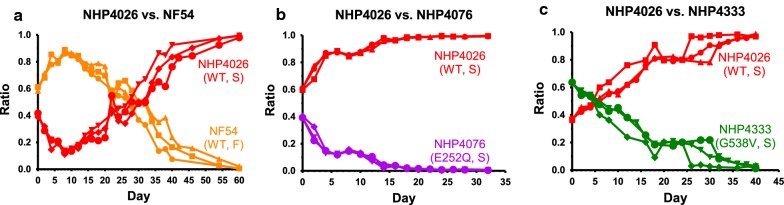
Parasite line competitive growth assays suggest a fitness advantage in the NHP4026 line. NHP4026, a slow clearance *kelch13*-wt isolate, was able to fully outcompete all seven isolates it was matched with in pairwise competitions, suggesting a potential fitness advantage. In NHP4026 versus NF54 (*kelch13*-wt, fast clearance), NF54 initially held a greater ratio of parasitaemia, but NHP4026 overcame NF54 around day 30 and fully outcompeted NF54 by day 60 (n = 3; **a**). NHP4026 outcompeted NHP4076 (*kelch13* E252Q, slow clearance) by day 24 (n = 3; **b**), and outcompeted NHP4333 (*kelch13* G538V, slow-clearance) by day 40 (n = 3; **c**)

NHP4026 (*kelch13*-wt, slow clearance, 7 wins, 0 losses) outcompeted even NF54, a line that has been used in the laboratory for many years. NF54 initially predominated but at day 30 a switch took place and NHP4026 fully outcompeted NF54 by day 60 (n = 3; Fig. 3a). NHP4026 also fully outcompeted NHP4076 (*kelch13* E252Q, slow clearance, 5 wins, 2 losses) by day 24, and NHP4333 (*kelch13* G538V, slow clearance, 4 wins, 3 losses) by day 40 (n = 3; Fig. 3b, c). Additionally, NHP4026 fully outcompeted NHP1337 (*kelch13* C580Y, slow clearance, 3 wins, 4 losses), NHP4302 (*kelch13*-wt, fast clearance, 2 wins, 5 losses), NHP3032 (*kelch13* K438N, fast clearance, 0 wins, 0 losses, 1 tie), and NHP4373 (*kelch13*-wt, slow clearance, 0 wins, 0 losses, 1 tie) (Additional file 6). Interestingly, NHP4026, while resistant to artemisinin, does not carry a resistance-associated coding mutation on the *kelch13* propeller domain (or elsewhere in the protein).

### *kelch13* E252Q mutant line displays a fitness advantage over other *kelch13* mutant lines

**Fig. 4:**
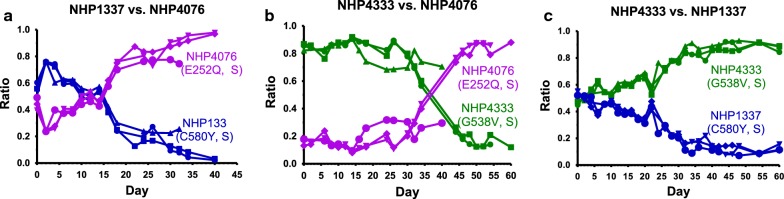
The Art-R associated *kelch13* E252Q allele shows a greater competitive fitness than other *kelch13* mutations. NHP4076 (E252Q) was competed against two other *kelch13* mutants, NHP1337 (C580Y) and NHP4333 (G538V). NHP4076 outcompeted NHP1337 by day 40 (n = 3; **a**) and NHP4333 by day 60 (n = 3; **b**). NHP4076 versus NHP4333 was set up unintentionally with NHP4076 only composing 20% of the total parasitaemia, yet was still able to outcompete NHP4333, giving reason to include this competition in the dataset. NHP4333 was also able to outcompete NHP1337 by day 40 (n = 3; **c**). The data suggests a competitive growth advantage of E252Q associated resistance over G538V and C580Y resistances, and a potential fitness disadvantage of C580Y resistance

Three slow clearance lines with different *kelch13* SNPs associated with resistance were included in the competitive growth assays: NHP4076 (*kelch13* E252Q), NHP4333 (*kelch13* G538V), and NHP1337 (*kelch13* C580Y). NHP4076 outcompeted NHP1337 by day 40 and NHP4333 by day 60 (Fig. 4a, b). The competition of NHP4076 versus NHP4333 was unintentionally initiated at a ratio of 0.8:0.2 NHP4333: NHP4076; nevertheless, NHP4076 outcompeted NHP4333 despite this initial parasitaemia deficit, NHP4333 outcompeted NHP1337 by day 40 (Fig. 4c).

By using genetically distinct lines recently isolated from patients in Southeast Asia, a fitness hierarchy can be generated for relevant parasites collected from a hot-spot of emerging drug resistance; for example, the E252Q mutant (NHP4076) outcompeted the G538V mutant (NHP43333) and the isolate with the C580Y mutant NHP1337. Notably, these parasites differ by more than their *kelch13* sequences; their genome-wide context, including the particular *kelch13* local haplotype, comprise the ‘genetic background’ of these parasite lines. These genetic backgrounds carry additional genetic determinants of resistance and compensation that may impact both the level of Art-R and the overall cost of fitness. In this study, the particular genetic background of the C580Y *kelch13* mutant that was isolated in 2011 from a patient in Thailand [32] exhibits the higher relative fitness cost when compared to the other two *kelch13* mutants in this study. A different C580Y-carrying genome emerged in 2008 in Western Cambodia and has outcompeted other haplotypes and by 2015 had spread to Thailand and Southern Laos, indicative of a selective sweep [20]; the assay developed here will be well-suited to determine fitness relationships among a series of C580Y parasites representing independent evolutionary origins of Art-R in a wide range of genetic backgrounds [36].

## Discussion

This is the first study to precisely quantify the relative fitness of a panel of natural parasite isolates collected from a hotspot of emerging drug resistance. By building on methods described in previous studies that focused on head-to-head competitions of experimentally modified lines, e.g. to directly test the effect of a point mutation in a controlled genetic background [25–27], this optimized assay is a platform that can be readily scaled to examine fitness relationships among large numbers of genetically diverse parasite lines.

Using this assay, natural isolates derived from SE Asia were unambiguously ranked. Among these, a C580Y mutant is not more fit than other *kelch* mutants, presumably due to the genome-wide context in which it resides. Moreover, based on the time and geographic region of parasite collection, our approach can address the specific conditions in which mutations arose and spread. For example, E252Q is a strong competitor in this study, consistent with the observation that this parasite was an early successful line in this geographic region [19]. Furthermore, a marked competitive advantage of an Art-R line, NHP4026, was observed that does not contain a coding mutation in K13. Together, these results raise new, testable questions and illustrate the potential usefulness of this assay to directly test hypotheses about origins and spread of specific mutations in specific genetic backgrounds to effectively predict and act to thwart the spread of resistance.

Knowledge of the fitness relationships of co-infecting, genetically distinct *P. falciparum* parasite lines will inform an understanding of the genetic structure of parasite populations, including the distribution of drug resistant lines and their propensities to expand in frequency and geographic range. Limited investigations of multi-clone infections of Art-R parasites have focused on the competitive fitness of specific *kelch13* mutations in experimentally controlled isogenic genetic backgrounds. This study describes and validates a plate-based, in vitro, competitive growth methodology and use it to determine fitness relationships among cloned parasite lines recently isolated from patients in Southeast Asia with a range of Art-R-associated *kelch13* alleles.

Experimental competitive growth studies of *Plasmodium* parasites in vitro have been limited to a few pairwise competitions to discern growth relationships among a few (two or three) parasite lines [7, 8, 15, 16] due to the high volume requirements of the methods used to determine the ratio of the competing parasite lines. However, by adapting the use of a straight-from-blood PCR kit and microsatellite markers to differentiate between two competing parasite lines, the throughput of competitive growth assays is increased (Additional file 4) compared to previous methods. Straight-from-blood PCR decreases the total culture volume for sample analysis (1 μl of culture); consequently, assays are conducted in 96-well plates, which decreases the amount of reagents, the number of cell cycles, and time/space requirements as well as the need for DNA extraction. The use of MS that have been used for genotyping for 20 years [29] allowed to easily and accurately distinguish between competing parasite lines. It is feasible using this method to catalogue head-to-head fitness relationships between larger numbers of parasites.

Each pairwise competition among seven genetically distinct parasite isolates and the NF54 strain was carried out for 60 days or until one line predominated. Win/loss records were used to determine each parasite’s relative fitness. The outcomes were transitive, allowing for an unambiguous ranking of these lines; this result indicates that it will be possible to infer relative competitive growth for large numbers of parasite lines by conducting a small subsampling of all possible direct competitions. The most competitively fit lines in this study (NHP4026 and NF54) fully outcompeted all other lines (with NHP4026 also fully outcompeting NF54). In other cases, such as for the mid-range NHP1337 (C580Y, Art-R) versus NHP4302 (*kelch13*-wt, Art-R) the winning parasite did not always fully replace its competitor over 60 days.

This study is the first to use a panel of seven isolates and the NF54 strain and conduct all-on-all pairwise competitions between them. This yielded transitive outcomes and an unambiguous ranking of competitive growth success, creating a robust and reproducible phenotype that has potential to be modeled and predicted. The transitivity of the competition outcomes could be in part due to direct differences in the proliferation rates of the individual lines. However, it is unlikely such simple growth relationships are the only factors at play. For example, non-linear growth relationships resulting from kin discrimination, wherein parasites recognize genetically dissimilar parasites and adjust to surrounding cues [37], quorum sensing, a mechanism by which parasites use signal molecules to elicit changes in gene expression that affect behaviour [38, 39], as well as parasite communications mitigated by release of exosome-like vesicles capable of delivering molecules and genes from infected RBCs [40] can all be revealed by these growth relationships.

Different competitive fitness levels of cultured parasites show the impact of different natural genotypes (i.e. drug resistance genes in their natural genetic backgrounds) on relative in vitro growth rates. This measure of fitness, while assessing relative costs of resistance to physiology and growth, does not directly measure the transmission of sexual stages to the mosquito vector. However, the experimental rigor afforded by this assay makes in vitro competitive growth a useful surrogate for biological fitness (transmission) that may predict tendencies of drug resistant isolates to spread in natural populations. With the emergence of Art-R parasites, tools to anticipate and counteract their expansion are urgently needed [14, 41].

The high competitive fitness of NHP4026 reported here is intriguing because this is the slowest-clearing parasite line out of the seven isolates and the NF54 strain that were tested (Table 1). The Art-R status was confirmed using the standard in vitro ring-stage survival assay (RSA) (Additional file 7) of this parasite that lacks a *kelch13* coding SNP [17]. Slow-clearing *kelch13* wild-type parasites have been reported previously [13, 23, 24, 42]; however, based on reports of increasing prevalence Art-R associated *kelch13* polymorphisms, it would have been expected that *kelch13* SNPs that are Art-R (NHP4333, NHP1337, NHP4076) would be more fit than *kelch13* wild-type resistant lines, with *kelch13* wild-type susceptible parasites being the most fit of all. However, strikingly different outcomes were observed than were expected.

All three *kelch13* Art-R mutant lines in this study outcompeted two Art-susceptible lines (NHP4302 and NHP3032) from the same geographic region; these Art-R lines also outcompeted a further *kelch13* wild-type, Art-R line, NHP4373. These results demonstrate that *kelch13* status of each parasite line alone does not predict relative fitness outcomes and point to the importance of understanding the components of their genetic backgrounds, co-segregating compensatory mutations which have varying capacities to abrogate the fitness costs observed of Art-R mutations [19]. Interestingly, wild-type *kelch13* Art-R parasites were both the most and least competitively fit parasites in this pilot study. It is interesting to consider whether our in vitro observation of a highly fit Art-R *kelch13* wild-type parasite (NHP4026) is being observed in the field. The relative frequency of wild-type *kelch13* is decreasing areas of Southeast Asia [43], however data is lacking regarding the resistance status of these parasites. Because there is no marker for non-*kelch13* Art-R, this form of resistance would escape easy detection.

The *kelch13* mutations examined in this paper include a line with a C580Y-encoding SNP in the propeller domain, which is where most SNPs reside in Art-R parasites [13, 21]. The C580Y line used for this study is less competitively fit than the parasite isolates carrying E252Q and G538V, a finding that diverges from the general prediction that C580Y would prevail because this *kelch13* mutation has expanded dramatically in frequency and distribution under drug pressure in Southeast Asia [36]. A possible explanation for the less competitively fit C580Y line relative to the E252Q and G538V lines is a suboptimal genetic background of this lineage.

The E252Q mutation lies outside of the K13 propeller region, consistent with the highest fitness cost being associated with mutations in the propeller domain, which could potentially explain why the E252Q line is more competitively fit relative to the parasite isolates carrying the C580Y and G538V. Many different haplotypes including C580Y *kelch13* have been associated with Art-R in Thailand, Cambodia, Vietnam, Laos, and Myanmar [44], but only one C580Y haplotype lineage, which emerged in 2008 in Western Cambodia, has outcompeted other haplotypes and spread to Thailand and southern Laos, indicative of a selective sweep [20]. This particular C580Y line was isolated from a Thai patient in 2007, from a time and place in which C580Y mutants were very rare amongst artemisinin-resistant parasites [36]. E252Q was the predominant SNP at the time that the samples utilized in this study were collected [19], however, since 2010, the prevalence of C580Y and other SNPs has surpassed E252Q in this area [45]. This could suggest that this particular C580Y line isolated in 2007 had not yet accumulated the necessary compensatory mutations (i.e. genetic background) required for competitively fitness.

Distinct population genetic structures distinguish these regions across a relatively small geographic area; consequently, it is likely that the different genetic backgrounds have played important roles in the fitness and spread of Art-R parasites. The Thailand–Myanmar border region from which parasites for this study were isolated differs from the Cambodia–Laos–Thailand cluster of Art-R populations and also those from the China–Myanmar region. Therefore, it is also possible that the relatively low competitive fitness of the C580Y line in our study could be due to its lineage (*kelch13* haplotype along with its genome-wide background) that differs from the one that has spread across Western Cambodia and Thailand.

Regardless of the exact reason for the fitness cost of the C580Y line used in this study, several recent studies have corroborated the findings from this study. A recent study comparing experimentally modified isogenic lines that differed only for the *kelch13* mutation determined that C580Y carries a greater competitive fitness burden than R561H compared directly in the same genetic background [26]. However, using a similar approach, but in a different genetic background than that used by Nair et al. [26], Straimer et al. [25] demonstrated that C580Y conveys significantly less fitness cost compared to either R539T or I543T. These seemingly conflicting study results could potentially be explained by the presence or absence of compensatory mutations in the genetic backgrounds.

Genome-wide association studies are demonstrating the importance of gene combinations and complex genetic architecture of Art-R with the assumption that compensatory mutations are co-evolving with resistance [22, 36]. However, discerning secondary resistance loci and loci involved in artemisinin partner drug resistance from fitness loci will require direct measures of fitness as outlined here. As an alternative, measuring fitness and drug phenotypes in the progeny of experimental genetic crosses between unique parasite lines can quantify the contribution of causal and compensatory loci to drug resistance phenotypes and fitness. This optimized in vitro competitive growth assay will allow us to use this approach to determine the genetic basis of the highly competitively fit NHP4026 parasite, including the components of fitness compensation pathways. The transitive nature of this phenotype measured using the optimized competitive growth assay will allow us to determine the relative fitness phenotypes of each of the recombinant progeny of a recent genetic cross involving NHP4026 × NF54-HT-GFP-luc to identify quantitative trait loci (QTL) [30]. The optimization of this assay to 96-well plates and the transitive nature of the phenotype makes population and linkage studies feasible, for example to dissect (and compare) genetic backgrounds of independently evolved Art-R *kelch13* lineages.

Finally, the competitive growth experiments in this study were carried out in the absence of artemisinin drug pressure; future competitive growth studies will also include artemisinin drug pressure. This approach could highlight the extent of competitive release, in which resistant isolates, ordinarily outcompeted by sensitive parasites, overcome the fitness deficit in the presence of drug [7–9], as well as the possibility that set of fitness relationships among Art-R parasites varies depending on the presence of drug.

## Conclusions

Through the design and application of an optimized in vitro competitive growth assay, differences in competitive fitness of seven genetically distinct *P. falciparum* lines from Southeast Asia, both artemisinin-sensitive and resistant, were elucidated. It was determined that a *kelch13* wild-type, resistant line had the highest competitive fitness and that regional differences in the genetic structure of parasite populations may account for differences in the fitness of *kelch13* SNP associated resistant lines, as the results showed the C580Y resistant line to be the least fit Art-R *kelch13* SNP line. This methodology will allow the high-throughput implementation of competitive growth experiments to help understand the spread of artemisinin-resistant markers in populations and guide targeted therapy against infections.

## Supplementary information

**Additional file 1.** Table of microsatellite markers used for DNA Amplification and CEQ Fragment Analysis. (A) The four microsatellite markers used in this study, their forward and reverse sequences, annealing temperatures (Tm), and the CEQ fragment sizes for each parasite line used in this study to determine relative densities of various parasite lines in the mixed cultures. (B) A matrix of the specific microsatellite marker used to determine the relative densities of two parasite lines in a competition. For example, the microsatellite marker TA119 was used to determine the relative densities of NF54 and NHP4026 in competition.

**Additional file 2.** Quantification of parasite density using CEQ fragment analysis. DNA samples amplified using primers for microsatellite markers were analysed with CEQ fragment analysis to determine the experimentally observed “measured” relative density ratio of each parasite in each of the known mixtures. The “measured” ratios were compared to the known (expected) “calculated” relative density ratios and plotted to create a standard curve for two parasites. This was repeated for a total of three repetitions. The standard curve (with a slope of 1.010 ± 0.01551 and an r^2^ value of 0.9914) shows that this method of microsatellite marker analysis using fluorescent primers and fragment analysis can accurately measure a wide range of parasite densities. It also determined that DNA from a second parasite cloned line does not have any significant impact on the ability to quantify the relative density of parasite DNA from the first parasite cloned line.

**Additional file 3.** Consistency of competition outcomes between biological replicates of NHP4026 versus NF54. The throughput of this optimized competitive growth assays makes it much easier to set up technical replicates, and it also makes it easier to set up biological replicates. Four separate competitions (four biological replicates) of the NF54 versus NHP4026 competition were set up with three technical replicates per biological replicate. Dotted lines at y = 0.05 and y = 0.95 show when the winner of the competition was declared, and the competition was ended. (A) In every biological replicate, NHP4026 outcompeted NF54. The only differences between the biological replicates were the starting ratios (due to variability in counting parasitaemia to start competitions) and the day in which one isolate reached 95% of the population, ending the competition. Biological replicate 1 (NHP4026_1 and NF54_1) took ~ 54 days to complete, biological replicate 2 took ~ 20 days to complete, while biological replicates 3 and 4 took ~ 32 and ~ 38 days, respectively. (B) All biological replicates (with their corresponding technical replicates) were averaged and the mean was plotted along with the standard error. In all replicates, NHP4026 outcompetes NF54.

**Additional file 4.** Timeline of optimized competition growth assays. The improved throughput of this optimized 96-well plate assay allows thawing, synchronization, and set up of one biological replicate with three technical replicates of competitions between eight parasites (28 competitions) in approximately 10 days. Additional biological replicates can be set up using left over culture grown for an additional life cycle from the first biological replicate or new cultures can be thawed and set up in competitions in approximately 10 days. The time and reagents required for maintenance of plates is greatly reduced by the reduced volume compared to flask methods, allowing for multichannel pipetting for the dilution of parasitaemia and the decreased space required to store plates compared to flasks. The analysis of samples is made simpler and quicker by allowing samples to be easily stored until researchers want to analyse the samples, not requiring a DNA extraction, only using 1 μl of whole culture for the PCR, and a simple dilution and sample preparation to run samples on the CEQ for fragment analysis. While each competition might take a variable amount of time to win (when one parasite line reaches 95% of the population), all-on-all competitions of 28 parasites with three technical replicates and analysis can be done in 31–73 days.

**Additional file 5.** Consistency between 5 ml and 96-well plate competition assays. In the competition P3 versus NF54, NF54 outcompeted P3 by day 22 in 5 ml flasks (A) and 96-well plates (B). P2 outcompeted P3 by day 32 in 5 ml flasks (C) and by day 42 in 96-well plates (D). NF54 outcompeted NHP1337 by day 22 in both 5 ml flasks (E) and 96-well plates (F). These confirm the reliability of 96-well plate competitive growth assay methodology.

**Additional file 6.** Parasite line win/loss records order lines by competition outcomes. Each parasite line was competed against all seven other parasite lines, and win/loss records were determined for each and used to order lines from highest competitive fitness to lowest fitness. NHP4026 (7-0 record) had complete wins over all seven other parasite lines. The least fit parasite lines, NHP4373 and NHP3032, were unable to outcompete any other parasite line and had records of 0 wins, 0 losses, 1 tie. Middle-range fitness parasite lines (NHP4076, NHP4333, NHP1337, and NHP4302) had records that were a mix of complete and partial wins.

**Additional file 7.** Ring-stage survival assay percent proliferation values for NHP4026, NHP1337, and NF54 as an in vitro metric to confirm resistant and sensitive isolates. The ring-stage survival assays from 0 to 3 h were carried out as previously described with minor modifications [28]. 72 h after drug treatment, slides were made on all cultures and 5000 RBCs were counted per culture. Percent proliferation was measured by the percent parasitaemia in the DHA treated culture over the percent parasitaemia in DMSO treated cultures. Each isolate has two biological replicates each with three technical replicates and the mean is graphed with the standard deviation. Parasites are considered resistant if the percent proliferation is greater than 5% [13]; the cutoff is marked as a dotted line on the graph.

## Data Availability

Data can be made available upon request to the corresponding author.

## Abbreviations

CQ-S: chloroquine-sensitive
CQ-R: chloroquine-resistant
Art-R: artemisinin resistant
SNPs: single-nucleotide polymorphisms
ICM: incomplete media
RBC: red blood cells
IDC: intraerythrocytic development cycle
DHA: dihydroartemisinin
DMSO: dimethyl sulfoxide
CEQ: Capillary Electrophoretic (CE) Genetic Analysis System (CEQ)
WT: wild-type
P3: parasite 3
QTL: quantitative trait loci

## References

[1] Lenski RE (1997). The cost of antibiotic resistance—from the perspective of a bacterium. Ciba Foundation Symp..

[2] Babiker HA (2009). Seasonal fluctuation of drug-resistant malaria parasites: a sign of fitness cost. Trends Parasitol..

[3] Hughes D, Andersson DI (2015). Evolutionary consequences of drug resistance: shared principles across diverse targets and organisms. Nat Rev Genet.

[4] Childs LM, Buckee CO (2015). Dissecting the determinants of malaria chronicity: why within-host models struggle to reproduce infection dynamics. J R Soc Interface.

[5] Abkallo HM, Tangena JA, Tang J, Kobayashi N, Inoue M, Zougrana A (2015). Within-host competition does not select for virulence in malaria parasites; studies with *Plasmodium yoelii*. PLoS Pathog..

[6] McGuigan L, Callaghan M (2015). The evolving dynamics of the microbial community in the cystic fibrosis lung: CF microbiome. Environ Microbiol.

[7] Wacker MA, Turnbull LB, Walker LA, Mount MC, Ferdig MT (2012). Quantification of multiple infections of *Plasmodium falciparum* in vitro. Malar J..

[8] Hayward R, Saliba KJ, Kirk K (2005). *pfmdr1* mutations associated with chloroquine resistance incur a fitness cost in *Plasmodium falciparum*. Mol Microbiol..

[9] Bushman M, Morton L, Duah N, Quashie N, Abuaku B, Koram KA (2016). Within-host competition and drug resistance in the human malaria parasite *Plasmodium falciparum*. Proc Biol Sci..

[10] Lewis IA, Wacker M, Olszewski KL, Cobbold SA, Baska KS, Tan A (2014). Metabolic QTL analysis links chloroquine resistance in *Plasmodium falciparum* to impaired hemoglobin catabolism. PLoS Genet.

[11] Gabryszewski SJ, Modchang C, Musset L, Chookajorn T, Fidock DA (2016). Combinatorial genetic modeling of *pfcrt*-mediated drug resistance evolution in *Plasmodium falciparum*. Mol Biol Evol.

[12] Petersen I, Gabryszewski SJ, Johnston GL, Dhingra SK, Ecker A, Lewis RE (2015). Balancing drug resistance and growth rates via compensatory mutations in the *Plasmodium falciparum* chloroquine resistance transporter: PfCRT impacts antimalarial resistance and growth rates. Mol Microbiol.

[13] Ashley EA, Dhorda M, Fairhurst RM, Amaratunga C, Lim P, Suon S (2014). Spread of artemisinin resistance in *Plasmodium falciparum* malaria. N Engl J Med.

[14] Wilairat P, Kümpornsin K, Chookajorn T (2016). *Plasmodium falciparum* malaria: convergent evolutionary trajectories towards delayed clearance following artemisinin treatment. Med Hypotheses.

[15] Pollitt LC, Huijben S, Sim DG, Salathé RM, Jones MJ, Read AF (2014). Rapid response to selection, competitive release and increased transmission potential of artesunate-selected *Plasmodium chabaudi* malaria parasites. PLoS Pathog.

[16] Hott A, Tucker MS, Casandra D, Sparks K, Kyle DE (2015). Fitness of artemisinin-resistant *Plasmodium falciparum* in vitro. J Antimicrob Chemother.

[17] Ariey F, Witkowski B, Amaratunga C, Beghain J, Langlois A-C, Khim N (2014). A molecular marker of artemisinin-resistant *Plasmodium falciparum* malaria. Nature.

[18] Fairhurst RM, Dondorp AM (2016). Artemisinin-resistant *Plasmodium falciparum* malaria. Microbiol Spectr..

[19] Anderson TJC, Nair S, McDew-White M, Cheeseman IH, Nkhoma S, Bilgic F (2017). Population parameters underlying an ongoing soft sweep in southeast Asian malaria parasites. Mol Biol Evol.

[20] Imwong M, Suwannasin K, Kunasol C, Sutawong K, Mayxay M, Rekol H (2017). The spread of artemisinin-resistant *Plasmodium falciparum* in the Greater Mekong subregion: a molecular epidemiology observational study. Lancet Infect Dis..

[21] Torrentino-Madamet M, Fall B, Benoit N, Camara C, Amalvict R, Fall M (2014). Limited polymorphisms in *k13* gene in *Plasmodium falciparum* isolates from Dakar, Senegal in 2012–2013. Malar J..

[22] Cerqueira GC, Cheeseman IH, Schaffner SF, Nair S, McDew-White M, Phyo AP (2017). Longitudinal genomic surveillance of *Plasmodium falciparum* malaria parasites reveals complex genomic architecture of emerging artemisinin resistance. Genome Biol.

[23] Boullé M, Witkowski B, Duru V, Srprawat K, Nair S, McDew-White M (2016). Artemisinin-resistant *Plasmodium falciparum* K13 mutant alleles, Thailand–Myanmar border. Emerg Infect Dis..

[24] Mukherjee A, Bopp S, Magistrado P, Wong W, Daniels R, Demas A (2017). Artemisinin resistance without *pfkelch13* mutations in *Plasmodium falciparum* isolates from Cambodia. Malar J..

[25] Straimer J, Gnädig NF, Stokes BH, Ehrenberger M, Crane AA, Fidock DA (2017). *Plasmodium falciparum**k13* mutations differentially impact ozonide susceptibility and parasite fitness in vitro. mBio.

[26] Nair S, Li X, Arya GA, McDew-White M, Ferrari M, Nosten F (2018). Fitness costs and the rapid spread of *kelch13*-C580Y substitutions conferring artemisinin resistance. Antimicrob Agents Chemother.

[27] Straimer J, Gnädig NF, Witkowski B, Amaratunga C, Duru V, Ramadani AP (2015). K13-propeller mutations confer artemisinin resistance in *Plasmodium falciparum* clinical isolates. Science.

[28] Witkowski B, Khim N, Chim P, Kim S, Ke S, Kloeung N (2013). Reduced artemisinin susceptibility of *Plasmodium falciparum* ring stages in western Cambodia. Antimicrob Agents Chemother.

[29] Su X (1999). A genetic map and recombination parameters of the human malaria parasite *Plasmodium falciparum*. Science.

[30] Vaughan AM, Pinapati RS, Cheeseman IH, Camargo N, Fishbaugher M, Checkley LA (2015). *Plasmodium falciparum* genetic crosses in a humanized mouse model. Nat Methods.

[31] WorldWide Antimalarial Resistance Network (WWARN). Parasite Clearance Estimator. http://www.wwarn.org/tools-resources/toolkit/analyse/parasite-clearance-estimator-pce. Accessed 19 Dec 2018.

[32] Cheeseman IH, McDew-White M, Phyo AP, Sriprawat K, Nosten F, Anderson TJC (2015). Pooled sequencing and rare variant association tests for identifying the determinants of emerging drug resistance in malaria parasites. Mol Biol Evol.

[33] Phyo AP, Nkhoma S, Stepniewska K, Ashley EA, Nair S, McGready R (2012). Emergence of artemisinin-resistant malaria on the western border of Thailand: a longitudinal study. Lancet.

[34] Taylor AR, Schaffner SF, Cerqueira GC, Nkhoma SC, Anderson TJC, Sriprawat K (2017). Quantifying connectivity between local *Plasmodium falciparum* malaria parasite populations using identity by descent. PLoS Genet.

[35] Murray L, Stewart LB, Tarr SJ, Ahouidi AD, Diakite M, Amambua-Ngwa A (2017). Multiplication rate variation in the human malaria parasite *Plasmodium falciparum*. Sci Rep..

[36] Amato R, Pearson RD, Almagro-Garcia J, Amaratunga C, Lim P, Suon S (2018). Origins of the current outbreak of multidrug-resistant malaria in southeast Asia: a retrospective genetic study. Lancet Infect Dis..

[37] Reece SE, Drew DR, Gardner A (2008). Sex ratio adjustment and kin discrimination in malaria parasites. Nature.

[38] Atkinson S, Williams P (2009). Quorum sensing and social networking in the microbial world. Interface Focus..

[39] Dyer M, Day KP (2003). Regulation of the rate of asexual growth and commitment to sexual development by diffusible factors from in vitro cultures of *Plasmodium falciparum*. Am J Trop Med Hyg.

[40] Regev-Rudzki N, Wilson DW, Carvalho TG, Sisquella X, Coleman BM, Rug M (2013). Cell-cell communication between malaria-infected red blood cells via exosome-like vesicles. Cell.

[41] O’Brien C, Henrich PP, Passi N, Fidock DA (2011). Recent clinical and molecular insights into emerging artemisinin resistance in *Plasmodium falciparum*. Curr Opin Infect Dis..

[42] Sutherland CJ, Lansdell P, Sanders M, Muwanguzi J, van Schalkwyk DA, Kaur H (2017). Pfk13-independent treatment failure in four imported cases of *Plasmodium falciparum* malaria treated with artemether–lumefantrine in the United Kingdom. Antimicrob Agents Chemother.

[43] Kobasa T, Talundzic E, Sug-aram R, Boondat P, Goldman IF, Lucchi NW (2018). Emergence and spread of *kelch13* mutations associated with artemisinin resistance in *Plasmodium falciparum* parasites in 12 Thai provinces from 2007 to 2016. Antimicrob Agents Chemother.

[44] Ménard D, Clain J, Ariey F (2018). Multidrug-resistant *Plasmodium falciparum* malaria in the Greater Mekong subregion. Lancet Infect Dis..

[45] Phyo AP, Ashley EA, Anderson TJC, Bozdech Z, Carrara VI, Sriprawat K (2016). Declining efficacy of artemisinin combination therapy against *P. falciparum* malaria on the Thai–Myanmar border (2003–2013): the role of parasite genetic factors. Clin Infect Dis.

