# Supplementary material for: Pairwise growth competitions identify relative fitness relationships among artemisinin resistant Plasmodium falciparum field isolates
Source: Malar J. 2019 Aug 28;18:295. doi: 10.1186/s12936-019-2934-4 (PMC6714446; doi:10.1186/s12936-019-2934-4)
Supplement: Supplementary file 1 — Additional file 1. Table of microsatellite markers used for DNA Amplification and CEQ Fragment Analysis. (A) The four microsatellite markers used in this study, their forward and reverse sequences, annealing temperatures (Tm), and the CEQ fragment sizes for each parasite line used in this study to determine relative densities of various parasite lines in the mixed cultures. (B) A matrix of the specific microsatellite marker used to determine the relative densities of two parasite lines in a competition. For example, the microsatellite marker TA119 was used to determine the relative densities of NF54 and NHP4026 in competition. [file 12936_2019_2934_MOESM1_ESM.pdf]

A

MS marker	Forward Primer	Reverse Primer	Tm	CEQ Fragment Size for NHP4026	CEQ Fragment Size for NF54	CEQ Fragment Size for NHP4076	CEQ Fragment Size for NHP4333	CEQ Fragment Size for NHP1337	CEQ Fragment Size for NHP4302	CEQ Fragment Size for NHP4373	CEQ Fragment Size for NHP3032
**TA119**	TCCTCGATTATATTA TTGCA	TAATACATTCCCATT AGATG	53	223	241	235	229	230	238	230	230
**TA81**	TGGACAAATGGGAA AGGAT	TTTCACACAACACA GGATT	53	135	120	126	129	122	119	116	116
**TA77**	AAGCAAAAGAAAAT AAGGAA	GCACATAGTTGGTT CTCAC	53	197	197	197	-	-	197	197	206
**TA62**	ATTATAGTGAAGAT AGCCA	TTACTTTCATCACTA TTTG	48	164	173	164	-	164	164	164	164

B

	**NHP4026**						
**NF54**	TA119	**NF54**					
**NHP4076**	TA119	TA119	**NHP4076**				
**NHP4333**	TA119	TA119	TA119	**NHP4333**			
**NHP1337**	TA119	TA119	TA119	TA81	**NHP1337**		
**NHP4302**	TA119	TA62	TA81	TA119	TA119	**NHP4302**	
**NHP4373**	TA119	TA119	TA81	TA81	TA81	TA119	**NHP4373**
**NHP3032**	TA119	TA119	TA81	TA81	TA81	TA119	TA77
