# Supplementary figures and images for: Pairwise growth competitions identify relative fitness relationships among artemisinin resistant Plasmodium falciparum field isolates
Source: Malar J. 2019 Aug 28;18:295. doi: 10.1186/s12936-019-2934-4 (PMC6714446; doi:10.1186/s12936-019-2934-4)

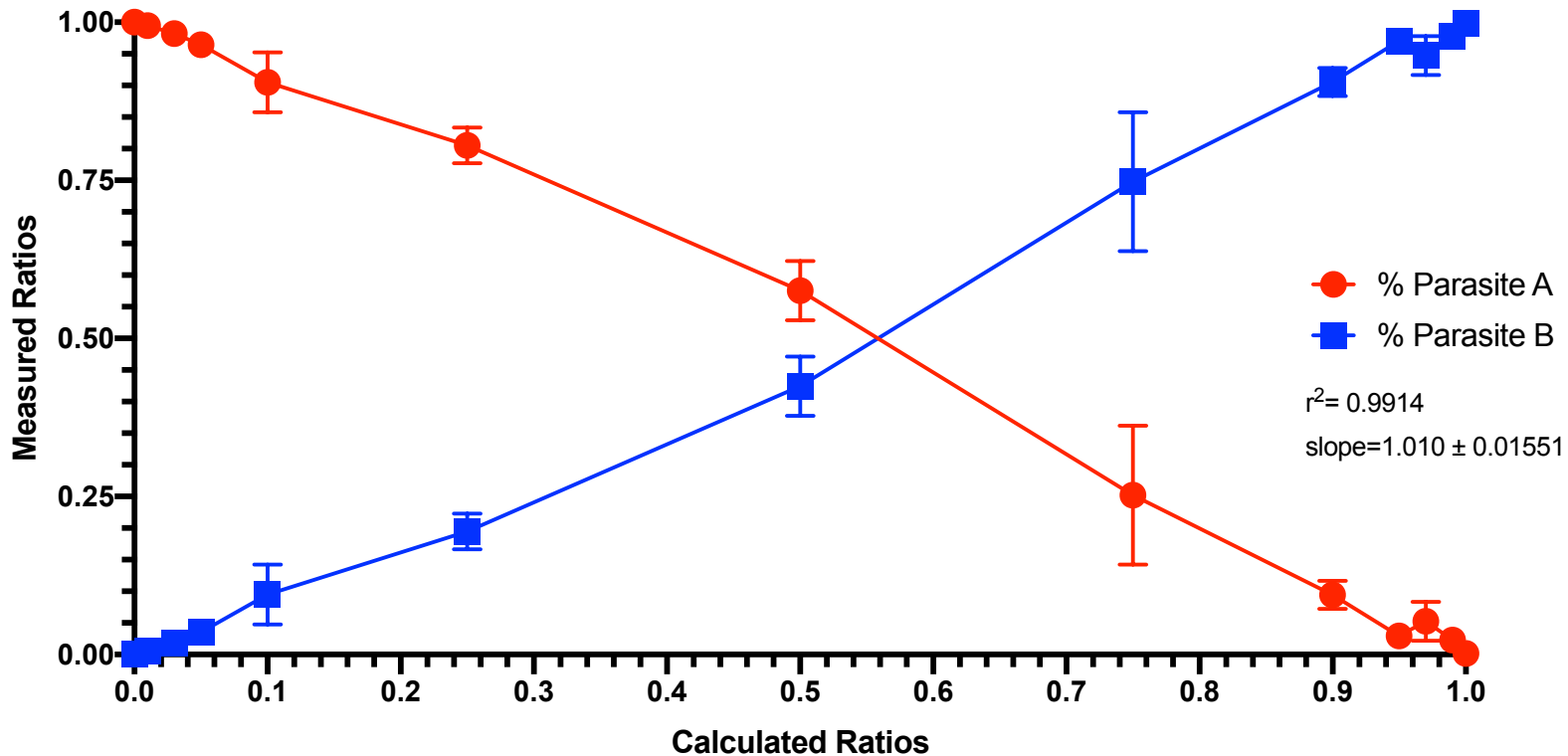

Supplement: Supplementary file 2 — Additional file 2. Quantification of parasite density using CEQ fragment analysis. DNA samples amplified using primers for microsatellite markers were analysed with CEQ fragment analysis to determine the experimentally observed “measured” relative density ratio of each parasite in each of the known mixtures. The “measured” ratios were compared to the known (expected) “calculated” relative density ratios and plotted to create a standard curve for two parasites. This was repeated for a total of three repetitions. The standard curve (with a slope of 1.010 ± 0.01551 and an r2 value of 0.9914) shows that this method of microsatellite marker analysis using fluorescent primers and fragment analysis can accurately measure a wide range of parasite densities. It also determined that DNA from a second parasite cloned line does not have any significant impact on the ability to quantify the relative density of parasite DNA from the first parasite cloned line. [file 12936_2019_2934_MOESM2_ESM.pdf]

## A NHP4026 vs. NF54: biological replicates

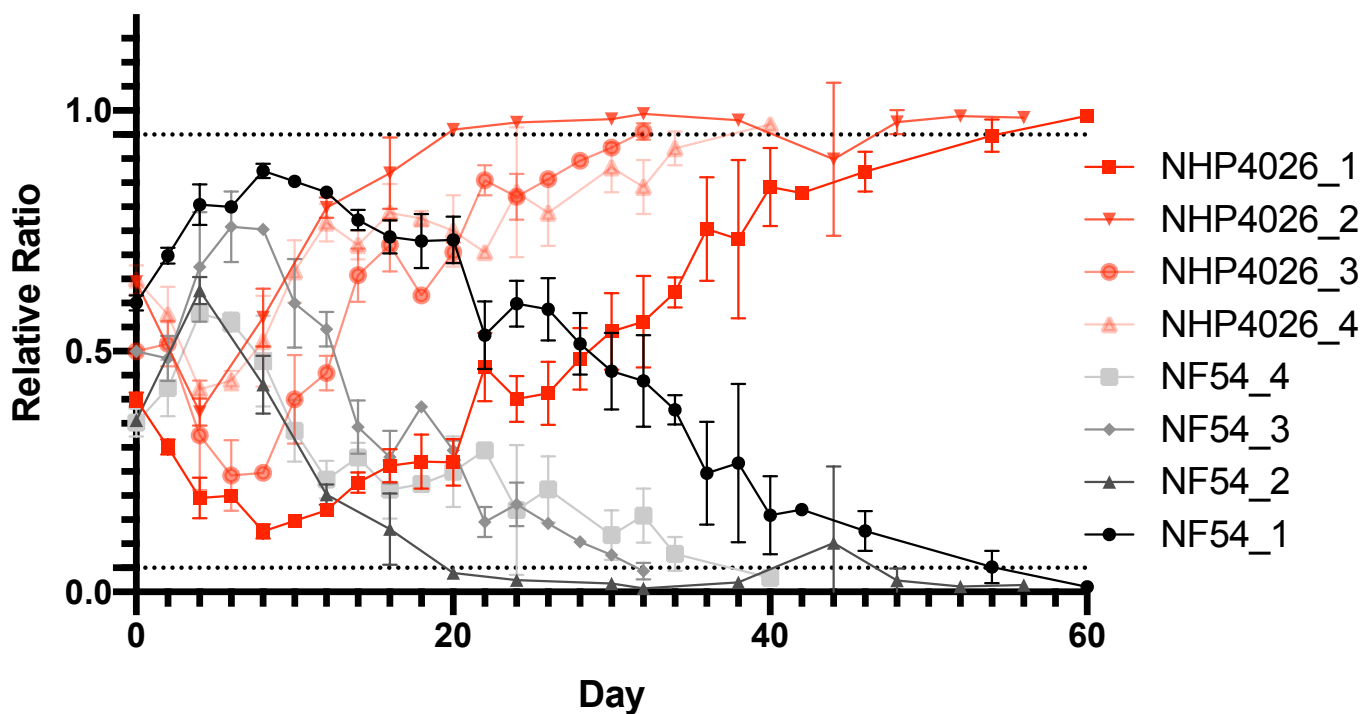

## B NHP4026 vs. NF54: means of biological replicates

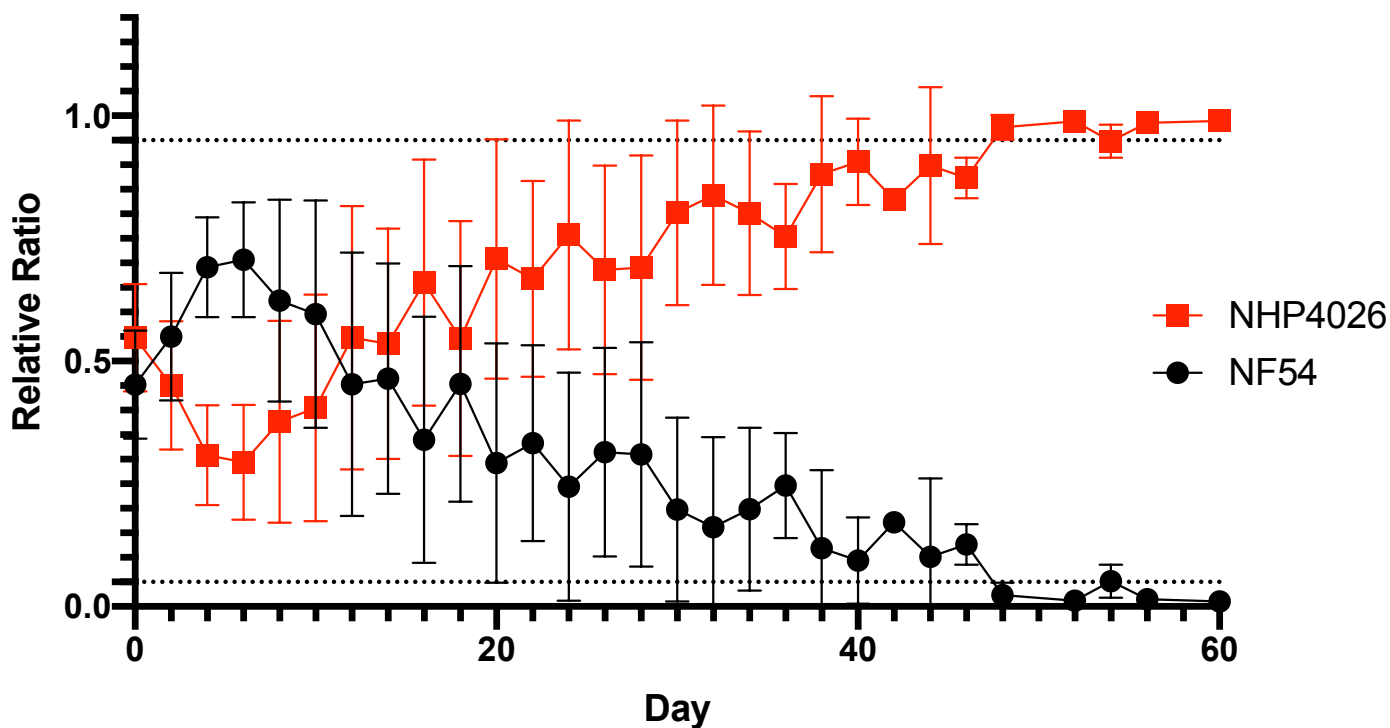

Supplement: Supplementary file 3 — Additional file 3. Consistency of competition outcomes between biological replicates of NHP4026 versus NF54. The throughput of this optimized competitive growth assays makes it much easier to set up technical replicates, and it also makes it easier to set up biological replicates. Four separate competitions (four biological replicates) of the NF54 versus NHP4026 competition were set up with three technical replicates per biological replicate. Dotted lines at y = 0.05 and y = 0.95 show when the winner of the competition was declared, and the competition was ended. (A) In every biological replicate, NHP4026 outcompeted NF54. The only differences between the biological replicates were the starting ratios (due to variability in counting parasitaemia to start competitions) and the day in which one isolate reached 95% of the population, ending the competition. Biological replicate 1 (NHP4026_1 and NF54_1) took ~ 54 days to complete, biological replicate 2 took ~ 20 days to complete, while biological replicates 3 and 4 took ~ 32 and ~ 38 days, respectively. (B) All biological replicates (with their corresponding technical replicates) were averaged and the mean was plotted along with the standard error. In all replicates, NHP4026 outcompetes NF54. [file 12936_2019_2934_MOESM3_ESM.pdf]

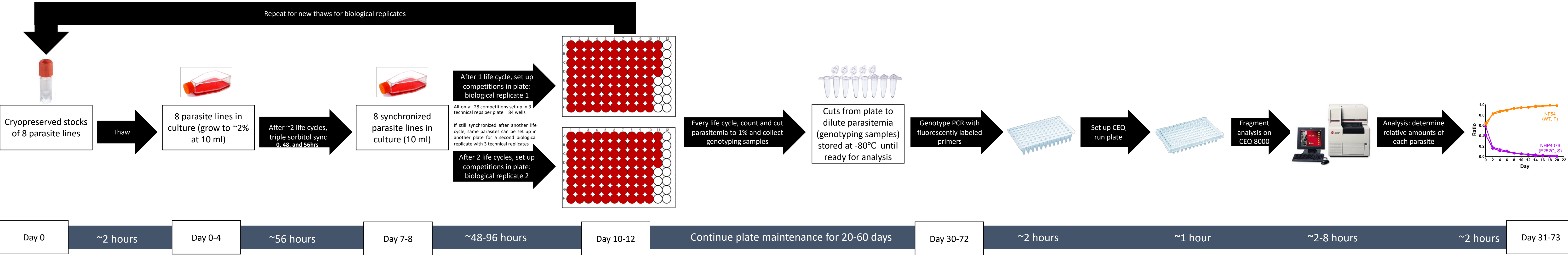

Supplement: Supplementary file 4 — Additional file 4. Timeline of optimized competition growth assays. The improved throughput of this optimized 96-well plate assay allows thawing, synchronization, and set up of one biological replicate with three technical replicates of competitions between eight parasites (28 competitions) in approximately 10 days. Additional biological replicates can be set up using left over culture grown for an additional life cycle from the first biological replicate or new cultures can be thawed and set up in competitions in approximately 10 days. The time and reagents required for maintenance of plates is greatly reduced by the reduced volume compared to flask methods, allowing for multichannel pipetting for the dilution of parasitaemia and the decreased space required to store plates compared to flasks. The analysis of samples is made simpler and quicker by allowing samples to be easily stored until researchers want to analyse the samples, not requiring a DNA extraction, only using 1 μl of whole culture for the PCR, and a simple dilution and sample preparation to run samples on the CEQ for fragment analysis. While each competition might take a variable amount of time to win (when one parasite line reaches 95% of the population), all-on-all competitions of 28 parasites with three technical replicates and analysis can be done in 31–73 days. [file 12936_2019_2934_MOESM4_ESM.pdf]

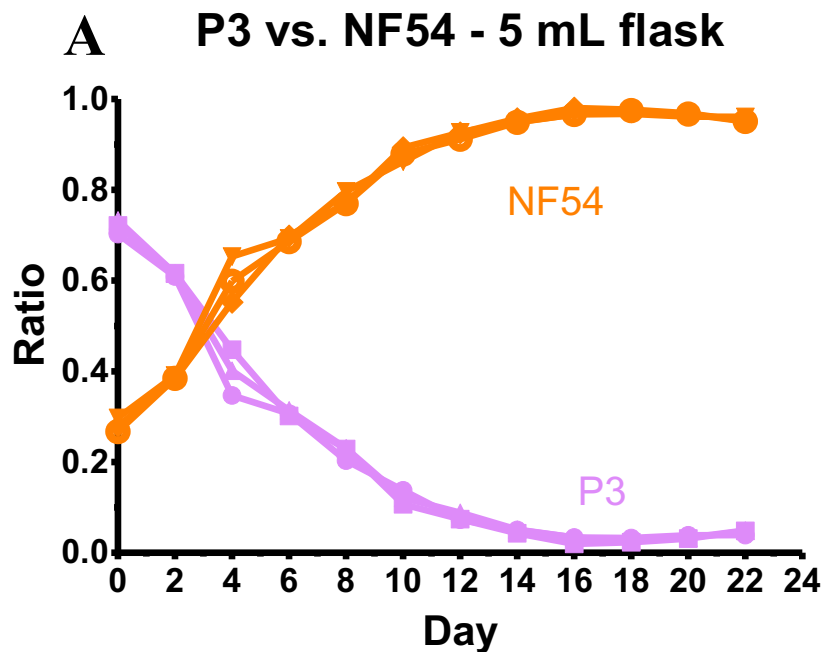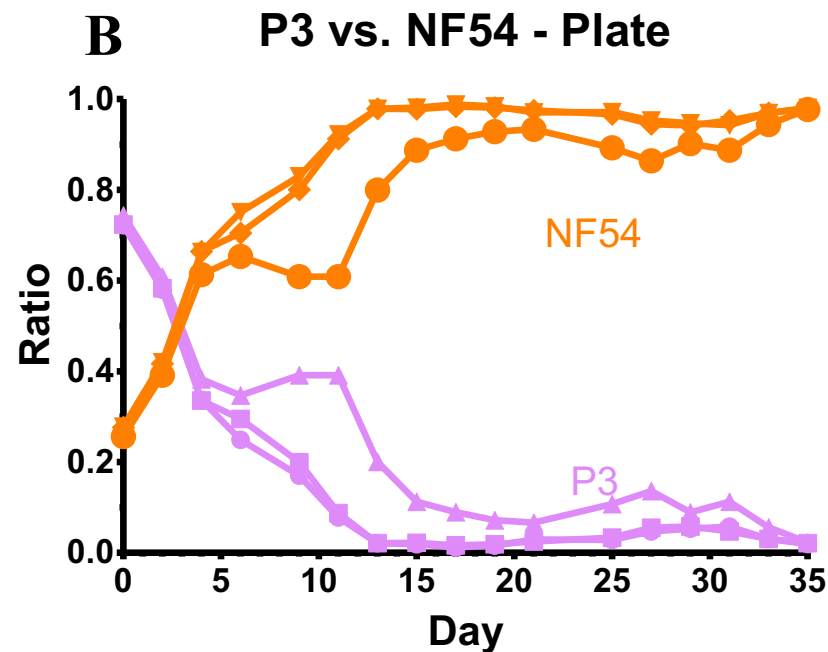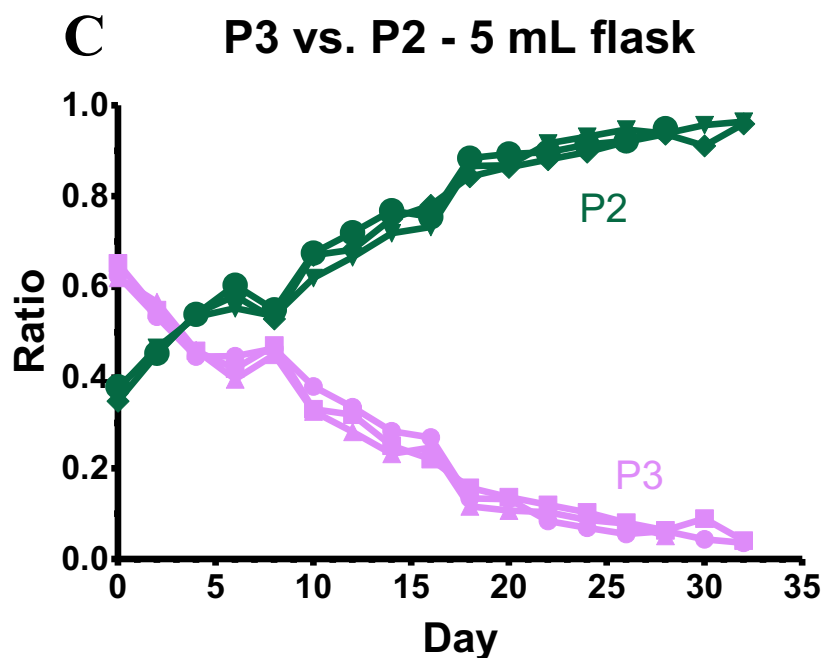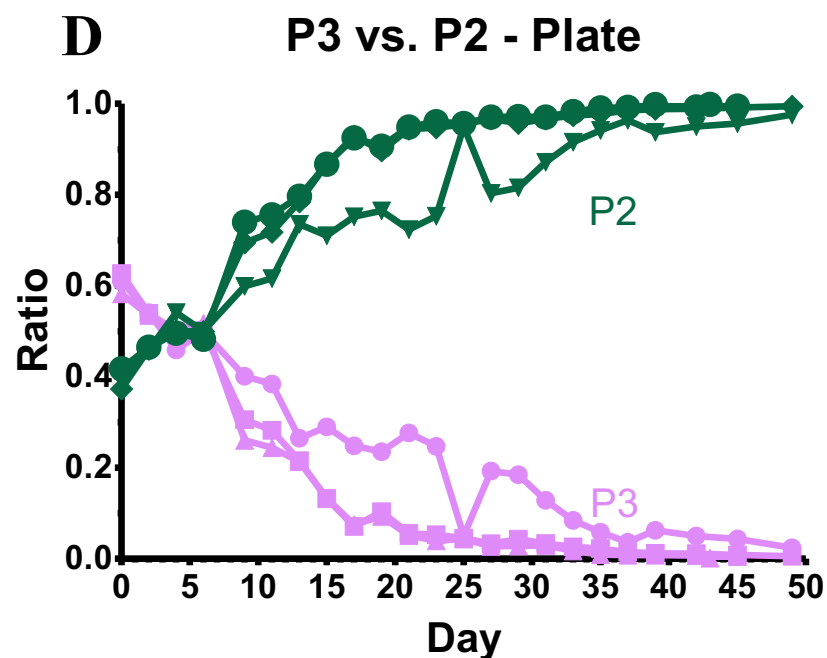

Supplement: Supplementary file 5 — Additional file 5. Consistency between 5 ml and 96-well plate competition assays. In the competition P3 versus NF54, NF54 outcompeted P3 by day 22 in 5 ml flasks (A) and 96-well plates (B). P2 outcompeted P3 by day 32 in 5 ml flasks (C) and by day 42 in 96-well plates (D). NF54 outcompeted NHP1337 by day 22 in both 5 ml flasks (E) and 96-well plates (F). These confirm the reliability of 96-well plate competitive growth assay methodology. [file 12936_2019_2934_MOESM5_ESM.pdf]

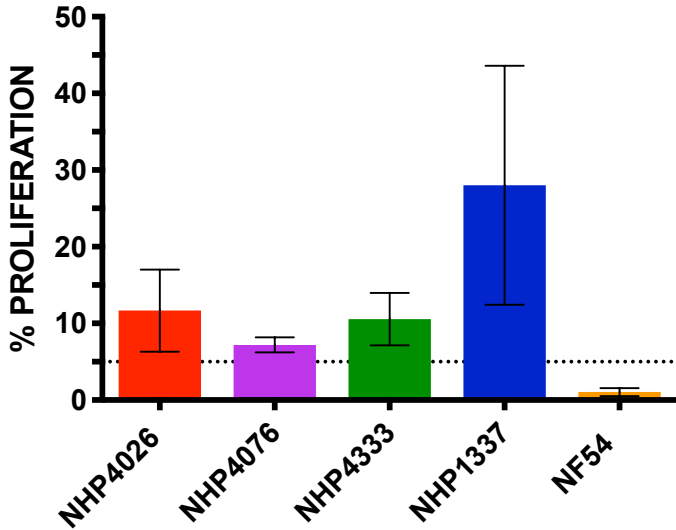

Supplement: Supplementary file 7 — Additional file 7. Ring-stage survival assay percent proliferation values for NHP4026, NHP1337, and NF54 as an in vitro metric to confirm resistant and sensitive isolates. The ring-stage survival assays from 0 to 3 h were carried out as previously described with minor modifications [28]. 72 h after drug treatment, slides were made on all cultures and 5000 RBCs were counted per culture. Percent proliferation was measured by the percent parasitaemia in the DHA treated culture over the percent parasitaemia in DMSO treated cultures. Each isolate has two biological replicates each with three technical replicates and the mean is graphed with the standard deviation. Parasites are considered resistant if the percent proliferation is greater than 5% [13]; the cutoff is marked as a dotted line on the graph. [file 12936_2019_2934_MOESM7_ESM.pdf]
