# Supplementary material for: Pairwise growth competitions identify relative fitness relationships among artemisinin resistant Plasmodium falciparum field isolates
Source: Malar J. 2019 Aug 28;18:295. doi: 10.1186/s12936-019-2934-4 (PMC6714446; doi:10.1186/s12936-019-2934-4)
Supplement: Supplementary file 6 — Additional file 6. Parasite line win/loss records order lines by competition outcomes. Each parasite line was competed against all seven other parasite lines, and win/loss records were determined for each and used to order lines from highest competitive fitness to lowest fitness. NHP4026 (7-0 record) had complete wins over all seven other parasite lines. The least fit parasite lines, NHP4373 and NHP3032, were unable to outcompete any other parasite line and had records of 0 wins, 0 losses, 1 tie. Middle-range fitness parasite lines (NHP4076, NHP4333, NHP1337, and NHP4302) had records that were a mix of complete and partial wins. [file 12936_2019_2934_MOESM6_ESM.pdf]

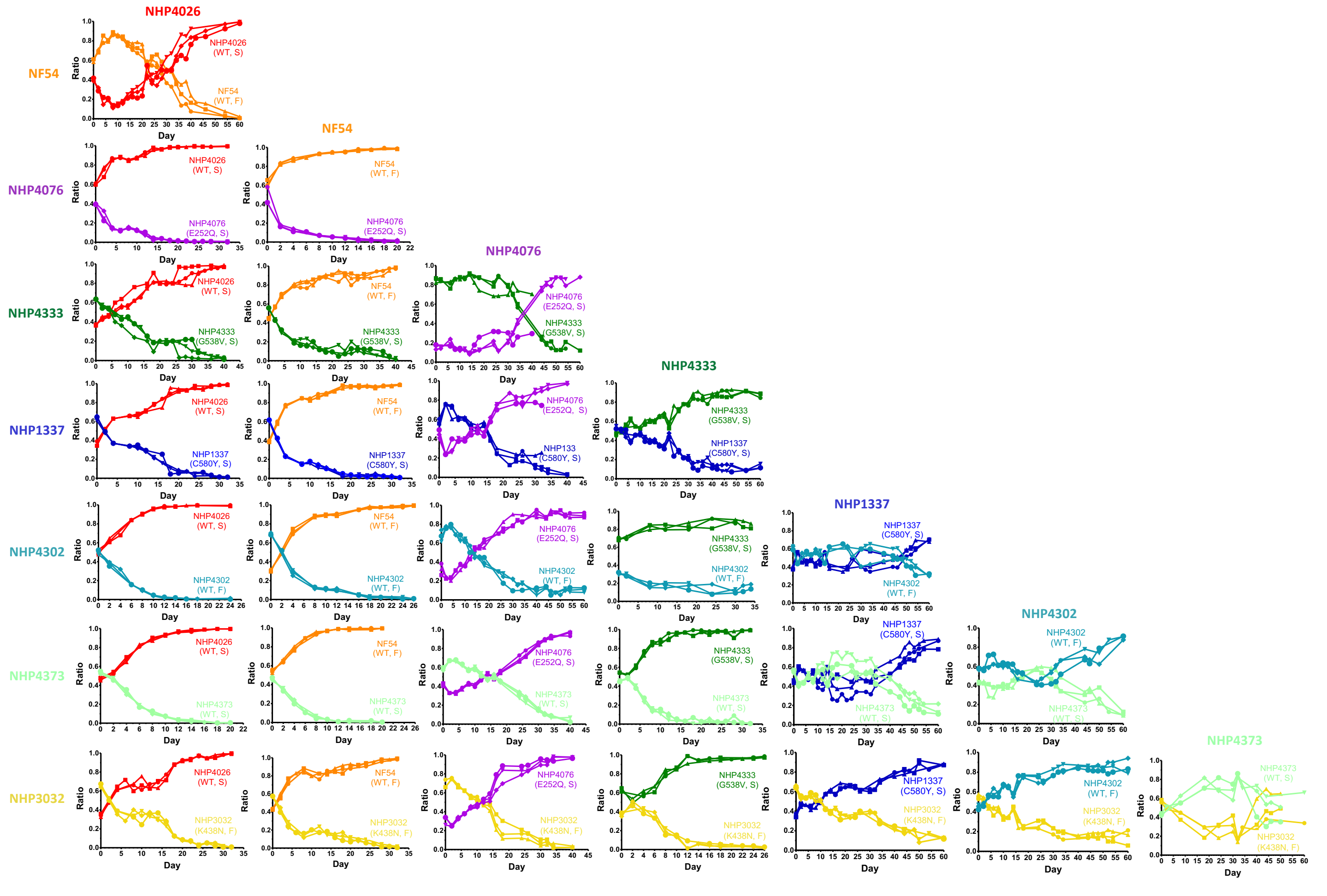
NHP4026
NF54
NHP4076
NHP4333
NHP1337
NHP4302
NHP4373
NHP3032
NHP4026 (WT, S)
NF54 (WT, F)
NHP4076 (E252Q, S)
NHP4333 (G538V, S)
NHP1337 (C580Y, S)
NHP133 (C580Y, S)
NHP4302 (WT, F)
NHP4373 (WT, S)
NHP3032 (K438N, F)
Ratio
Day
